# Hyperspectral imaging system for multiplexed cancer marker detection: design and clinical evaluation

**DOI:** 10.1117/1.JBO.31.7.076504

**Published:** 2026-07-09

**Authors:** Oren Ben-Ami, Shmoolik Mangan, Tal Keidar Haran, Raz Ben-Uri, Efrat Ofek, Yuval Garini, Leeat Keren, Iris Barshack, Boaz Brill

**Affiliations:** a Pentaomix Ltd., Rehovot, Israel; b Weizmann Institute of Science, Department of Molecular Cell Biology, Rehovot, Israel; c Hadassah Hebrew University Medical Center, Department of Pathology, Jerusalem, Israel; d Chaim Sheba Medical Center, Department of Pathology, Ramat Gan, Israel; e Technion − Israel Institute of Technology, Faculty of Biomedical Engineering, and Russell Berrie Nanotechnology Institute, Haifa, Israel

**Keywords:** hyperspectral imaging, Sagnac interferometer, multiplex immunofluorescence, immunohistochemistry, non-small cell lung cancer, diagnostic pathology, spectral unmixing

## Abstract

**Significance:**

Multiplexed biomarker assessment is increasingly required in oncologic diagnostics, yet tissue depletion from sequential immunohistochemistry (IHC) and molecular testing limits routine pathology. Many multiplex immunofluorescence platforms rely on iterative staining or complex instrumentation, restricting scalability and clinical adoption.

**Aim:**

The aim is to develop and clinically evaluate a hyperspectral multiplexed fluorescence imaging platform for simultaneous detection of multiple diagnostic biomarkers on a single formalin-fixed paraffin-embedded (FFPE) tissue section potentially compatible with routine workflows.

**Approach:**

A Fourier transform hyperspectral system incorporating a monolithic Sagnac interferometer was engineered for single-shot, full-spectrum acquisition without filter switching or iterative staining. A seven-biomarker lung cancer panel was applied in a single staining and imaging workflow, and custom spectral reconstruction and unmixing generated spatially resolved biomarker maps. Clinical performance was assessed retrospectively through blinded comparison with single-plex chromogenic IHC.

**Results:**

The system achieved submicron spatial resolution with minimal spectral cross-talk, enabling robust seven-channel imaging. Single-section analysis showed complete diagnostic concordance with reference IHC across the evaluated cohort of non-small cell lung cancer and other thoracic specimens and revealed biomarker co-localization, including tumor-associated PD-L1.

**Conclusions:**

This platform enables high-content diagnostic profiling from a single FFPE tissue section while conserving tissue and preserving spatial information, supporting further evaluation of its potential integration into routine clinical pathology workflows.

## 1 Introduction

Modern oncologic diagnostics increasingly rely on the integration of histopathology, immunohistochemistry (IHC), and molecular profiling to guide classification, prognostication, and treatment selection. Across many solid malignancies, this multi-modal diagnostic paradigm is constrained by the limited amount of tissue available from minimally invasive biopsies, which must support a growing number of sequential assays. As a result, tissue depletion has emerged as a systemic challenge in clinical pathology, contributing to incomplete diagnostic workups, delayed treatment decisions, and the need for repeat invasive procedures.^1^^,^^2^

In contemporary workflows, molecular testing, including next-generation sequencing (NGS), often consumes a substantial fraction of available biopsy material, frequently equivalent to dozens of histologic sections.^1^^,^^2^ This escalating tissue demand amplifies the need for diagnostic approaches that maximize information extraction per section, particularly for protein-level biomarkers that remain essential for lineage determination, predictive testing, and therapeutic stratification. Multiplex immunofluorescence (mIF) and related spatial imaging approaches have been developed to address this challenge by enabling simultaneous detection of multiple biomarkers within a single formalin-fixed paraffin-embedded (FFPE) tissue section while preserving spatial context at single-cell resolution.^3^^–^^10^ Such spatially resolved measurements provide access to biomarker co-expression patterns, tumor–immune interactions, and microenvironmental organization that cannot be reliably inferred from serial single-plex sections.^4^

Despite their analytical power, the clinical adoption of existing multiplexing platforms remains limited. Many approaches rely on iterative staining and imaging cycles, increasing acquisition time, susceptibility to antigen degradation, and computational burden associated with image registration and alignment.^11^ These factors impose significant workflow complexity and cost, limiting compatibility with routine clinical pathology laboratories that require robust, rapid, and scalable solutions.^5^^–^^10^

Hyperspectral imaging has emerged as a powerful modality for biomedical and cancer imaging applications and offers an alternative optical strategy for clinical multiplexing by capturing the full fluorescence emission spectrum at each spatial location without mechanical filter switching or iterative staining cycles.^12^^–^^15^ Specifically, Fourier-transform-based spectral imaging approaches enable rapid, photon-efficient acquisition with high spectral resolution and minimal cross-talk, making them well-suited both for disentangling spectrally overlapping fluorophores in dense multiplex panels and for supporting emerging oncology diagnostic applications.^13^^,^^16^^–^^20^

**Fig. 1:**
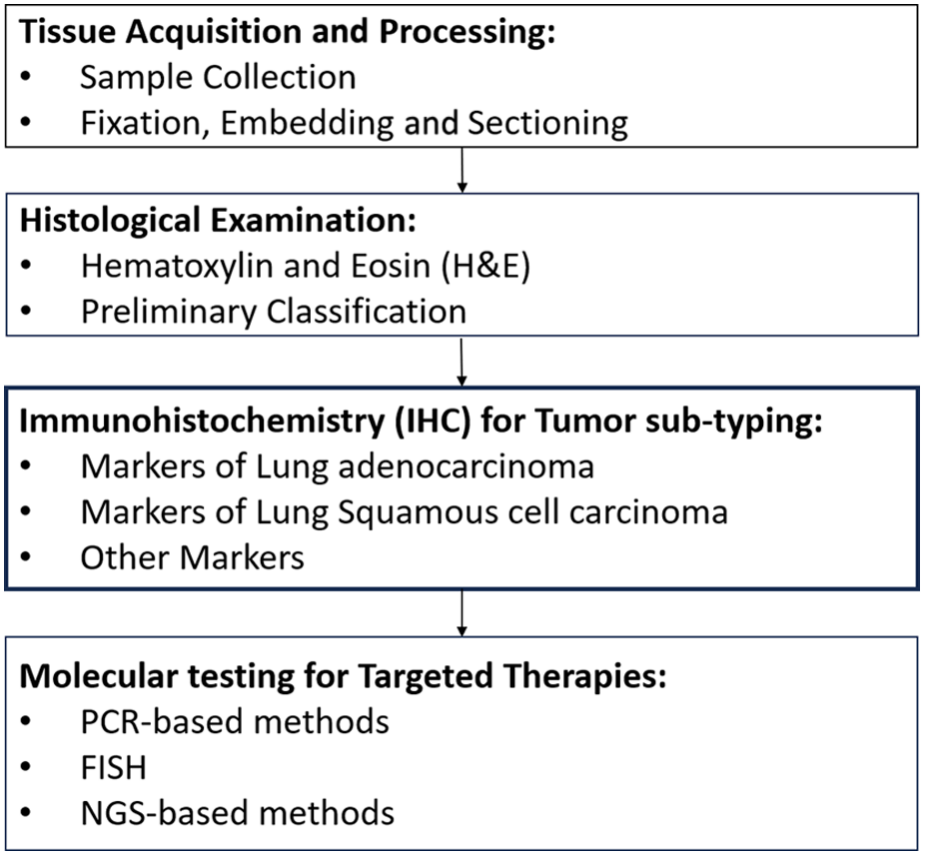
Diagnostic workflow in lung cancer and tissue utilization constraints. Schematic illustration of the standard diagnostic workflow for lung cancer, including histopathological evaluation, IHC, and molecular testing. All diagnostic steps are typically performed on the same limited tissue specimen, resulting in competition for scarce biopsy material and motivating the need for tissue-preserving multiplex approaches.

Here, we present a hyperspectral fluorescence imaging system developed with consideration for clinical applicability, incorporating a monolithic Sagnac interferometer and Fourier-based spectral reconstruction algorithms. This platform enables single-shot, full-spectrum acquisition of multiple fluorescent markers with high spatial and spectral fidelity while avoiding iterative staining cycles and complex optical filter exchanges. We demonstrate the diagnostic performance of this approach using non-small cell lung cancer (NSCLC) as a clinically relevant test case. Lung cancer remains the leading cause of cancer-related mortality worldwide, accounting for more than 1.8 million deaths annually, with NSCLC representing ∼85% of cases.^21^ NSCLC diagnosis relies on the integration of morphology and IHC markers such as pan-cytokeratin (PanCK), TTF1, and P40 for histologic subtyping, as well as additional predictive biomarkers including ALK and PD-L1 that guide targeted- and immuno-therapies.^22^^–^^29^ Figure 1 illustrates the standard lung cancer diagnostic workflow, highlighting the sequential use of histological examination, IHC, and molecular testing on the same limited tissue specimen.

In a retrospective NSCLC validation pilot study, we observed complete diagnostic concordance between diagnoses obtained using the proposed hyperspectral multiplexing approach and reference single-plex IHC results, supporting the potential of this system for tissue-efficient multiplex diagnostic workflows and motivating further clinical validation studies.

## 2 Materials and Methods

### 2.1 Tissue Staining Procedure

FFPE tissue sections were baked at 65°C for 20 min and subsequently incubated (2×5  min) in xylene at room temperature (RT) for deparaffinization. Tissue rehydration was performed through a series of 3-min RT incubations in the following ethanol solutions: two incubations in 100% EtOH, followed by one incubation in 90% EtOH and one incubation in 70% EtOH. This was followed by one incubation (3 min at RT) in distilled water and a 1× PBS incubation (3 min at RT), which was followed by an antigen retrieval step done in a pressure cooker using 1× TE buffer (pH 9.0; Abcam, Cambridge, United Kingdom; low pressure, for 20 to 30 min). Following a cooling period of at least 30 min to equilibrate samples to RT, endogenous peroxidase activity was quenched by incubation in 3% hydrogen peroxide for 15 to 30 min in RT. Following three 5-min washes in 1× PBS, tissue sections were incubated for 1 to 2 h in RT in a blocking solution composed of 10% goat serum, 6% bovine serum albumin (BSA), and unlabeled streptavidin in 1× PBS. A pre-incubation step with free-biotin was then performed to saturate tissue’s biotin-binding sites and prevent subsequent nonspecific binding (of biotin-conjugated primary antibody used downstream). Primary antibodies (see Table 1) were then applied at their optimal working dilutions and incubated under standard conditions (either 1 to 2 h at RT or overnight at 4°C in a humidified chamber).

**Table 1:** Biomarker panel composition for multiplex immunofluorescence analysis of non-small cell lung cancer.

Target	Subcellular localization	Antibody clone (origin)	Fluorophore used	Detection method
TTF1	Nucleus	8G7G3/1 (mouse)	PE-eFluor 610	Unconjugated primary antibody, biotin-Fab, and streptavidin conjugate
P40	Nucleus	BC28 (mouse)	PE-iFluor 710	Conjugated primary antibody
PanCK	Membrane and cytoplasm	AE1/AE3 (mouse)	PE-Cy5	Conjugated primary antibody
PD-L1	Membrane	22C3 (mouse)	CF405S	Unconjugated primary antibody, HRP-conjugated anti-mouse secondary, and CF405S-tyramide (tyramide signal amplification)
ALK	Cytoplasm	D5F3 (rabbit)	CF405L	Unconjugated primary antibody, HRP-conjugated anti-rabbit secondary, and CF405L-tyramide (tyramide signal amplification)
CD45	Membrane	2B11 + PD7/26 (mouse)	mFluor Violet 610	Conjugated primary antibody
Nuclear DNA	Nucleus	N/A	NucSpot 500/515	Conjugated staining reagent

Wash steps were performed using 1× PBS containing 0.05% Tween-20 (PBS-T), with 3×5  min washes at RT. Primary antibodies used in this study and their corresponding vendors were as follows: anti-P40 (Fortis Life Sciences, Boston, Massachusetts, United States), anti-TTF1 (Abcam), anti-PD-L1 (Agilent, Santa Clara, California, United States), anti-pan-cytokeratin (Biotium, Fremont, California, United States), anti-CD45 (Biotium), and anti-ALK (Cell Signaling Technology, Danvers, Massachusetts, United States). Nuclear staining was performed using NucSpot 500/515 (Biotium). Tyramide signal amplification (TSA) reagents were purchased from Biotium (see Table 1). To detect these primary antibodies, several methods were utilized. Briefly, when possible, biological targets were detected using fluorophore-conjugated primary antibodies conjugated in-house, using standard manufacturer protocols. This refers to monoclonal antibodies targeting P40, PanCK, and CD45, all conjugated in-house with the following antibody-dye conjugation kits: PE-iFluor 710 (AAT-Bioquest, Pleasanton, California, United States), PE-Cy5 (Abcam), and mFluor Violet 610 (AAT-Bioquest), respectively. To detect TTF1, we used a three-step streptavidin–biotin-based staining assay (see Table 1). To enable ultra-sensitive detection of PD-L1 and ALK proteins, biomarkers requiring high detection power, we made use of commercial TSA reagents^30^^–^^32^ optimized in-house (see Table 1). Briefly, following incubation with the unconjugated primary antibody, the sections were washed (1× PBS-T, 5 min at RT, ×3) and then incubated with species-specific horseradish peroxidase (HRP)-conjugated secondary antibody. Following washings, the sections were incubated with 5  μM tyramide-conjugate (CF405S for PDL-1; CF405L for ALK) in a $H_{2}O_{2}$-supplemented commercial TSA buffer (Biotium) for 20 to 25 min at RT followed by washings (1× PBS-T, 5 min at RT, ×3). Following nuclear staining, the slides were mounted and kept in a dark humid area till imaged.

### 2.2 Imaging System: Overview

**Fig. 2:**
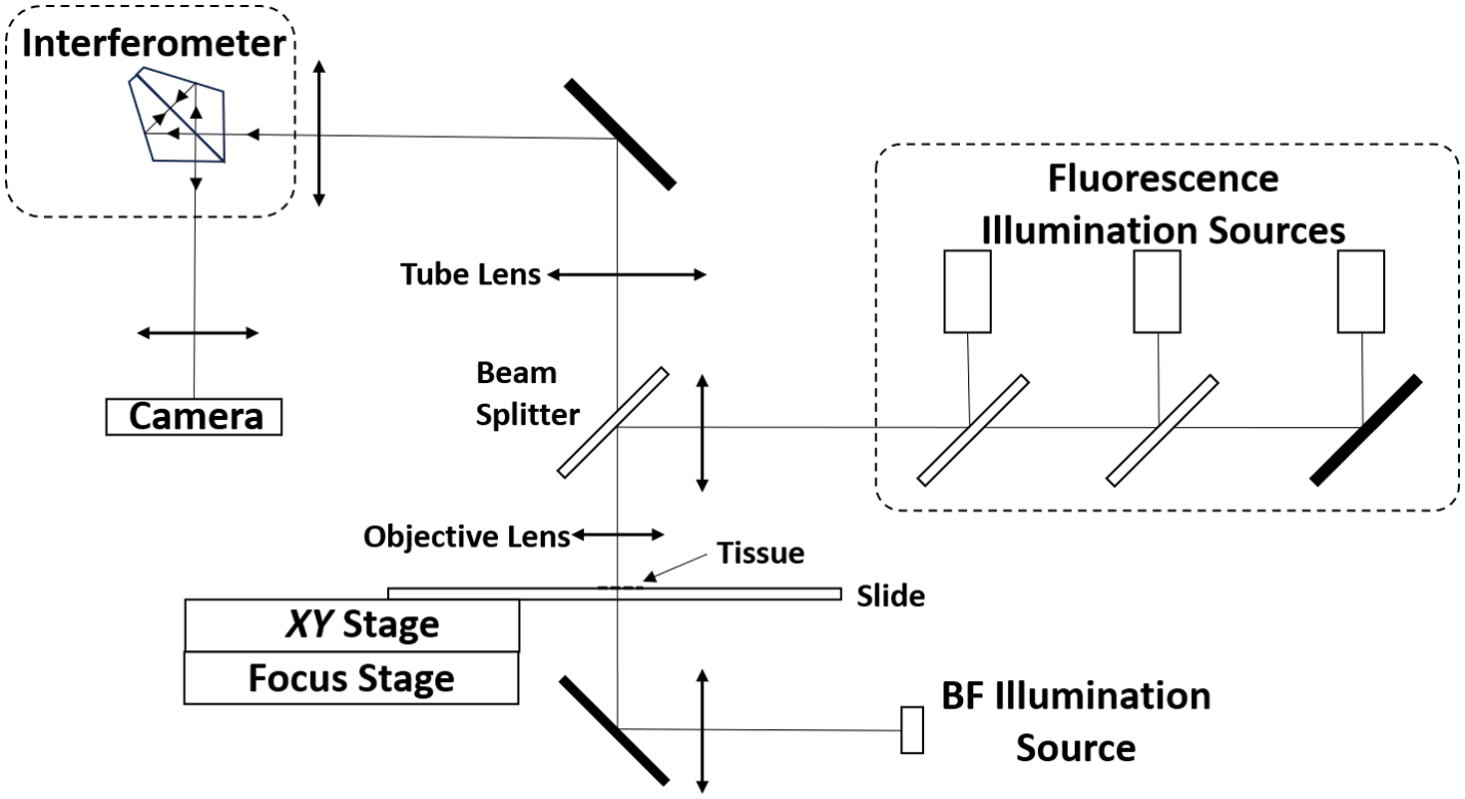
Optical architecture of the hyperspectral imaging system. Schematic overview of the hyperspectral fluorescence imaging system, illustrating the excitation paths, sample scanning stage, and detection arm incorporating a monolithic Sagnac interferometer. The interferometer introduces an angle-dependent optical path difference that encodes spectral information, enabling Fourier-transform-based reconstruction of fluorescence emission spectra without tunable filters or mechanically switched spectral elements.

The hyperspectral multiplex imaging system used in this study employs a Fourier-transform-based optical principle that enables acquisition of high-resolution spectral data without tunable filters, diffraction gratings, or mechanically switched spectral elements. Aside from the standard X−Y motorized scanning stage, the system contains no moving optical components, providing substantial benefits for clinical use, including improved mechanical robustness, spectral stability, and reduced maintenance requirements.^17^ As illustrated in Fig. 2, the core innovation of the system is the incorporation of a monolithic Sagnac interferometer into the fluorescence detection path.

Unlike Michelson-type interferometers, the Sagnac geometry introduces an angularly dependent optical path difference (OPD) between the clockwise and counter-clockwise propagation directions. For monochromatic light, this OPD produces a sinusoidal interference pattern whose spatial frequency varies predictably with wavelength. In the implemented optical layout, the interferometer is positioned within a collimated beam segment downstream of the tube lens. In this configuration, each ray’s angle encodes its lateral origin on the sample, allowing the interferometric modulation to encode spectral information while preserving spatial correspondence across the imaging field. After transmission through the interferometer, fluorescence emission is refocused onto a scientific Complementary Metal-Oxide-Semiconductor (sCMOS) camera, forming an image in which interference fringes are superimposed on the spatial fluorescence signal. For broadband fluorescence, each pixel records a weighted combination of sinusoidal components determined by the underlying emission spectrum and the interferometer’s angular-frequency response. As the sample is scanned laterally, each spatial location is measured under multiple OPD conditions, effectively sampling the Fourier domain of the emission spectrum. An inverse Fourier transform is applied to reconstruct the emission spectrum at each pixel, consistent with principles of Fourier transform spectral imaging.^16^^,^^18^^,^^33^

A major engineering advancement in this system is the use of a custom-fabricated monolithic Sagnac interferometer composed of two optically bonded precision prisms. Although this configuration does not provide tunability of mirror spacing, it offers markedly improved mechanical stability.

To support excitation of fluorophores with diverse absorption spectra, the system incorporates two high-power light-emitting diode (LED) illumination bands (405 and 490 nm). A multi-band dichroic beam splitter directs both excitation bands toward the sample while transmitting longer-wavelength fluorescence emission to the detection arm.

During acquisition, the sample is scanned in a raster pattern. At each stage position, images are sequentially acquired under both excitation LEDs. Between 80 and 120 positions along the X-axis are required (depending on the system configuration used) to adequately sample the Fourier domain for accurate spectral reconstruction. Although this generates multiple images per field of view, the system architecture does not rely on filter wheels, tunable components, or mechanically switched spectral elements—an intrinsic advantage of Fourier transform spectral imaging^34^ that may support simplified and mechanically stable spectral acquisition workflows.

The system hardware includes the following components:

• Illumination: two LED sources at 405 and 490 nm (LEDHub, Omicron Laserage, Rodgau-Dudenhofen, Germany)
• Objective lens: 20×, NA 0.8 fluorescence-optimized objective (Olympus, Tokyo, Japan)
• Detector: monochrome sCMOS camera (2464×2056  pixels, 16-bit; Lumenera LT-M2450, Ottawa, Canada) chosen for high quantum efficiency, low read noise, and fast frame rate
• Interferometer: custom monolithic Sagnac interferometer constructed from two optically bonded prisms.

The complete multiplex fluorescence imaging workflow consists of four stages:

1. Tissue staining: FFPE tissue sections were stained with a panel of primary antibodies and a nuclear counterstain, each paired to detection with a spectrally distinct fluorophore (see Sec. 2.1).
2. Hyperspectral acquisition: The stained mounted tissue section slides were scanned using the interferometer-based hyperspectral system to obtain raw interferometric image stacks.
3. Signal processing: raw data calibration and alignment, construction of Fourier vectors for each pixel, inverse Fourier transform, and spectral unmixing (see Sec. 2.3).
4. Image analysis: denoising, sharpening, cell segmentation, and biomarker-based classification.

Importantly, the number of reconstructed spectral values per pixel substantially exceeds the number of fluorophores in the multiplex assay. This overdetermined spectral representation improves the conditioning of the unmixing problem and enables reliable separation of fluorophores with partially overlapping spectra, surpassing the performance of multispectral systems that rely on a limited set of discrete spectral channels.

### 2.3 Imaging System: Calibration and Unmixing

To ensure optimal spectral reconstruction and quantitative fluorophore separation, several calibration procedures were implemented:

a. Flat-field calibration: An empty-field illumination acquisition was used to correct for pixel-to-pixel detection non-uniformity and spatial non-uniformity in the excitation profile.
b. Distortion calibration: High-precision optical targets were imaged to estimate and correct geometric distortion across the field of view, ensuring accurate spatial correspondence across the raster scan.
c. Stage motion calibration: The scanning stage was characterized to detect sub-pixel positional errors, and a correction vector was applied during reconstruction to maintain consistent OPD sampling across scan positions.
d. Spectral calibration: Narrow-band interference filters were imaged to determine the system transfer function and to refine the wavelength-to-Fourier frequency mapping used during inverse Fourier transform reconstruction.^35^^,^^36^

Tissue autofluorescence was modeled using a positive-only singular value decomposition least-squares fitting approach^37^^,^^38^ applied to matching sections of unstained control tissue, yielding tissue-specific autofluorescence components. Reference spectra for each fluorophore were obtained using a section of the relevant control tissue stained with a single fluorescent dye. For each pixel, the reconstructed emission spectrum was fit to a library consisting of fluorophore reference spectra and autofluorescence components. As multiple fluorophores can be excited by more than one illumination band, emission spectra from both excitation wavelengths were concatenated to form an extended spectral vector. Spectral unmixing was then performed in this extended spectral space using a constrained linear decomposition method.^39^^–^^41^

Standard signal processing procedures, including noise averaging, deconvolution-based sharpening, and exposure normalization, were applied prior to unmixing. Preliminary image analysis steps were also performed, including cell segmentation and biomarker-based classification, although these procedures were not essential for the core unmixing pipeline.

### 2.4 Retrospective Clinical Validation Pilot Study and Panel Design

To evaluate the system under clinically relevant conditions, a retrospective clinical validation pilot study was performed on NSCLC tissue specimens. Lung cancer was selected because it is one of the cancers where the scarcity of patient’s specimen represents a recognized diagnostic limitation in advanced disease, resulting in increased risk of incomplete biomarker assessment and likely adversely affected therapy selection.^2^

The study was performed under institutional review board approval (Hadassah Medical Center, protocol 250-23-HMO). Tissue samples from 16 clinical NSCLC cases and 4 thoracic non-NSCLC cases were selected from 2 previously constructed tissue microarrays (TMAs). Excision-based normal control tissues (thymus, lung, pancreas, and appendix) as well as lung cancer tissue sections were used to support assay development, panel optimization, and verification and validation.

The multiplex panel consisted of staining with a nuclear counterstain and a set of monoclonal primary antibodies targeting human TTF1, P40, PanCK, CD45, ALK, and PD-L1 (see Table 1 and Sec. 2.1). These biomarkers were chosen to address complementary diagnostic and therapeutic decision points, including the following: (a) identification of tumors of epithelial origin by utilization of the anti-PanCK antibody, detecting many cytokeratins; (b) initial NSCLC subtype assignment, differentiating adenocarcinoma (ADC) (commonly $\mathrm{TTF}1^{+}/P40^{-}$) from squamous cell carcinoma (SqCC) (commonly $P40^{+}/\mathrm{TTF}1^{-}$);^22^^,^^23^ (c) treatment selection for both PD-1/PD-L1-based immunotherapy and ALK-targeted therapy;^24^ and (d) visualization of the immune compartment and discrimination of tumor cell versus immune cell PD-L1 expression using the CD45 pan-hematopoietic lineage cell surface biomarker.

Each TMA slide was stained with the full multiplexed panel and imaged using the hyperspectral system. Cases underwent blinded pathology review, and diagnostic results were compared with historical immunohistochemical interpretations performed on a set of consecutive chromogenic IHC single-plex tissue section slides.

Clinically validated primary antibody clones were selected^25^ to ensure appropriate reference standards and clinical interpretability^25^ (see Table 1). Fluorophore and biological target pairing accounted for the anticipated co-localization, spectral separation, and expected expression levels of each target. For example, nuclear-associated biomarkers (TTF1 and P40) were separated by distinct emission spectra, a spectrally intermediate fluorophore was assigned to a non-nuclear biomarker, and fluorophores exhibiting superior imaging performance were assigned to the lowest expressing targets (and vice versa) to achieve balanced signal intensity across targets. Table 1 summarizes the fluorophores, detection methods, and antibody characteristics for each biomarker included in the multiplex panel.

### 2.5 Region of Interest (ROI)-Level Quantitative Comparison Analysis

For quantitative comparison between chromogenic IHC and mIF, ROI-level analyses were performed using histologically matched regions from consecutive clinical NSCLC tissue sections. Because identical cellular correspondence among consecutive sections was not expected, comparisons were conducted at the regional rather than single cell level. A total of 13 corresponding ROIs spanning high, intermediate, low, and negative staining regions were manually selected for each marker. Image analysis was performed using ImageJ (Fiji). For chromogenic IHC images, 3,3′-diaminobenzidine (DAB) signal intensity maps were extracted using hematoxylin-DAB (H-DAB) color deconvolution, inverted, and converted into 8-bit grayscale representations prior to ROI-based intensity measurements. Corresponding unmixed mIF channel images were similarly converted into 8-bit grayscale representations before intensity quantification. Pearson correlation coefficients and linear regression analyses were calculated among matched ROI intensity measurements.

### 2.6 Quantitative Spectral Cross-Talk Analysis in Clinical NSCLC Specimens

Quantitative assessment of potential spectral cross-talk under clinical tissue conditions was performed using a representative multiplexed NSCLC specimen exhibiting strong PanCK and P40 staining and reference-negative TTF1, PD-L1, and ALK staining. Representative tumor cells within the analyzed specimen co-expressed multiple biomarkers, including PanCK, P40, and the nuclear dye, thereby enabling assessment of potential spectral cross-talk under biologically relevant multiplex staining conditions. Cells were segmented in QuPath (v0.5.1) based on the nuclear staining channel, and per-cell nuclear and cytoplasmic mean intensity measurements were extracted from the unmixed fluorescence channels. During panel design, spectrally neighboring biomarkers were intentionally selected, where possible, to display distinct subcellular localization patterns, thereby enabling evaluation of potential spectral leakage while preserving clinically relevant biomarker combinations. Correlation analyses were performed among spectrally neighboring fluorophore channels, including cytoplasmic PanCK versus cytoplasmic P40, cytoplasmic PanCK versus cytoplasmic TTF1, and nuclear dye versus nuclear TTF1 signal intensities. Pearson correlation coefficients were calculated to evaluate potential signal co-variation among neighboring spectral channels. In addition, nuclear-to-cytoplasmic P40 intensity ratios were evaluated to assess preservation of expected nuclear localization patterns.

## 3 Results

### 3.1 Optical Performance

To evaluate the optical performance and spectral unmixing capabilities of the system, we first used commercial biotin-coated spherical polystyrene beads (5 µm diameter; Nanocs, New York, New York, United States), which provided a well-controlled test sample set. In this experiment, biotin-coated beads were divided into seven groups, each pre-stained with a single spectrally distinct streptavidin–fluorophore conjugate, yielding seven spectrally different populations of stained beads. These beads were then mixed in similar amounts, mounted on a slide, and imaged in our system, enabling a direct end-to-end assessment of the system’s spectral separation and channel cross-talk in a given system configuration.

**Fig. 3:**
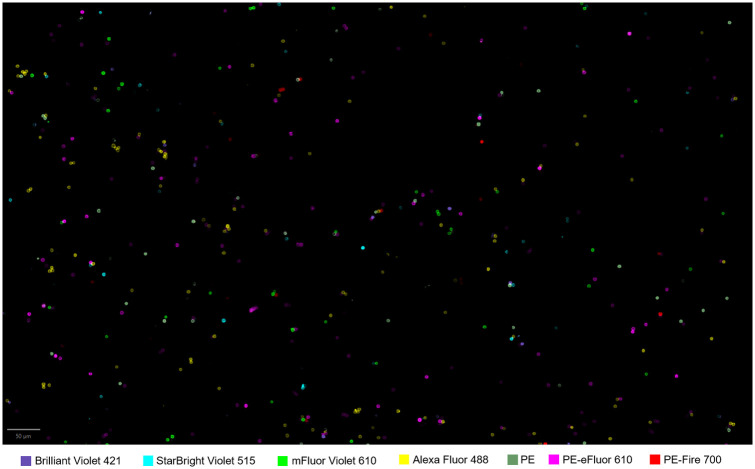
Optical performance assessment using single-color-labeled fluorescent beads. Pseudo-colored image of 5-μm-diameter spherical biotin-coated polystyrene beads, labeled with a single spectrally unique streptavidin–fluorophore conjugate selected from a collection of seven streptavidin dye conjugates (for dye names, see figure), mixed, mounted on a glass slide, and imaged in the system. Pseudo-colors shown represent the spectrally unmixed fluorophore assignments. This approach enables a controlled workflow to evaluate actual optical resolution and spectral cross-talk in a given system configuration. Scale bar: 50  μm.

A representative pseudo-colored multiplex image is shown in Fig. 3, where each color corresponds to one of seven fluorophores used for staining and included in the unmixing library.

As each bead was stained with a particular single fluorophore, the actual association of each bead primarily to a single channel supports the notion that the level of spectral cross-talk between any pair of channels is low and sufficient to enable unmixing of all fluorophores in this panel.

The spherical geometry of the beads also allowed evaluation of optical image quality and edge sharpness. Edge profile measurements indicated that the system’s lateral resolution was below the width of two pixels, corresponding to less than 0.66  μm under the imaging configuration reported here. In addition, regarding the system’s operational stability, system alignment and spectral reconstruction fidelity were maintained for months with no need for frequent recalibration, an essential property required for routine use in clinical settings.^40^

### 3.2 Retrospective Clinical Validation Pilot Study

**Fig. 4:**
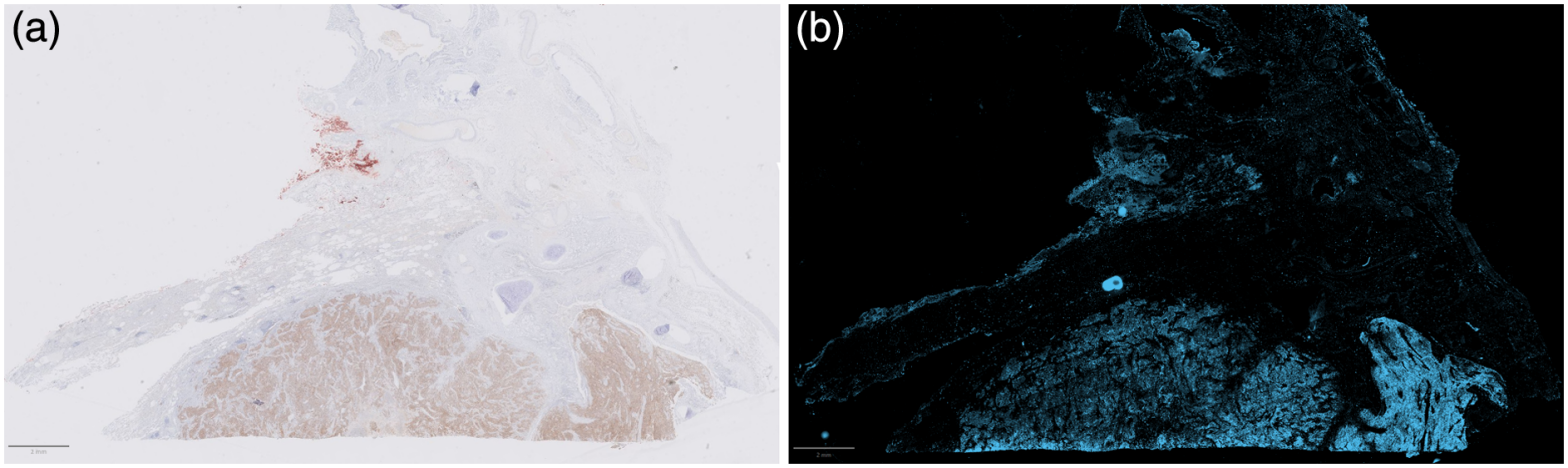
Large scanning area comparison of PD-L1 staining pattern observed in conventional IHC versus mIF. Representative low-magnification images of an NSCLC excision specimen stained for PD-L1, showing (a) standard single-plex chromogenic IHC and (b) mIF acquired using the hyperspectral imaging system. The images demonstrate similar large-scale PD-L1 staining patterns across the tissue section. Scale bar: 2 mm.

During assay development and verification, each IF assay was first optimized separately for its respective biological target using control tissues to confirm sufficient specificity, sensitivity, and robustness (see Supplemental Figs. S1 and S2). Multiplexed experiments were subsequently performed on control tissues to optimize and balance the multiplexed panel. Following mIF assay verification, initial clinical validation was performed using NSCLC tissue specimens previously characterized by standard single-plex chromogenic IHC in a collaborating pathology laboratory. These specimens were subsequently processed using the multiplexed staining protocol and imaged using the hyperspectral microscopy system, enabling direct comparison between the multiplexed and external reference clinical results. In contrast to synthetic calibration samples and control tissues, clinical NSCLC specimens present substantially greater complexity, including heterogeneous morphology, variable autofluorescence, and overlapping spectral signatures, thereby providing a stringent test of system performance. We first assessed the system’s capability to reproduce diagnostically relevant staining patterns over relatively large tissue areas. To this aim, we performed multiplexed panel staining on NSCLC excision tissue specimens and compared the results with a representative reference single-plex chromogenic IHC. Using PD-L1 as a representative marker, we show that our PD-L1 mIF channel data and the corresponding IHC section exhibit comparable staining patterns across the tissue (Fig. 4).

**Fig. 5:**
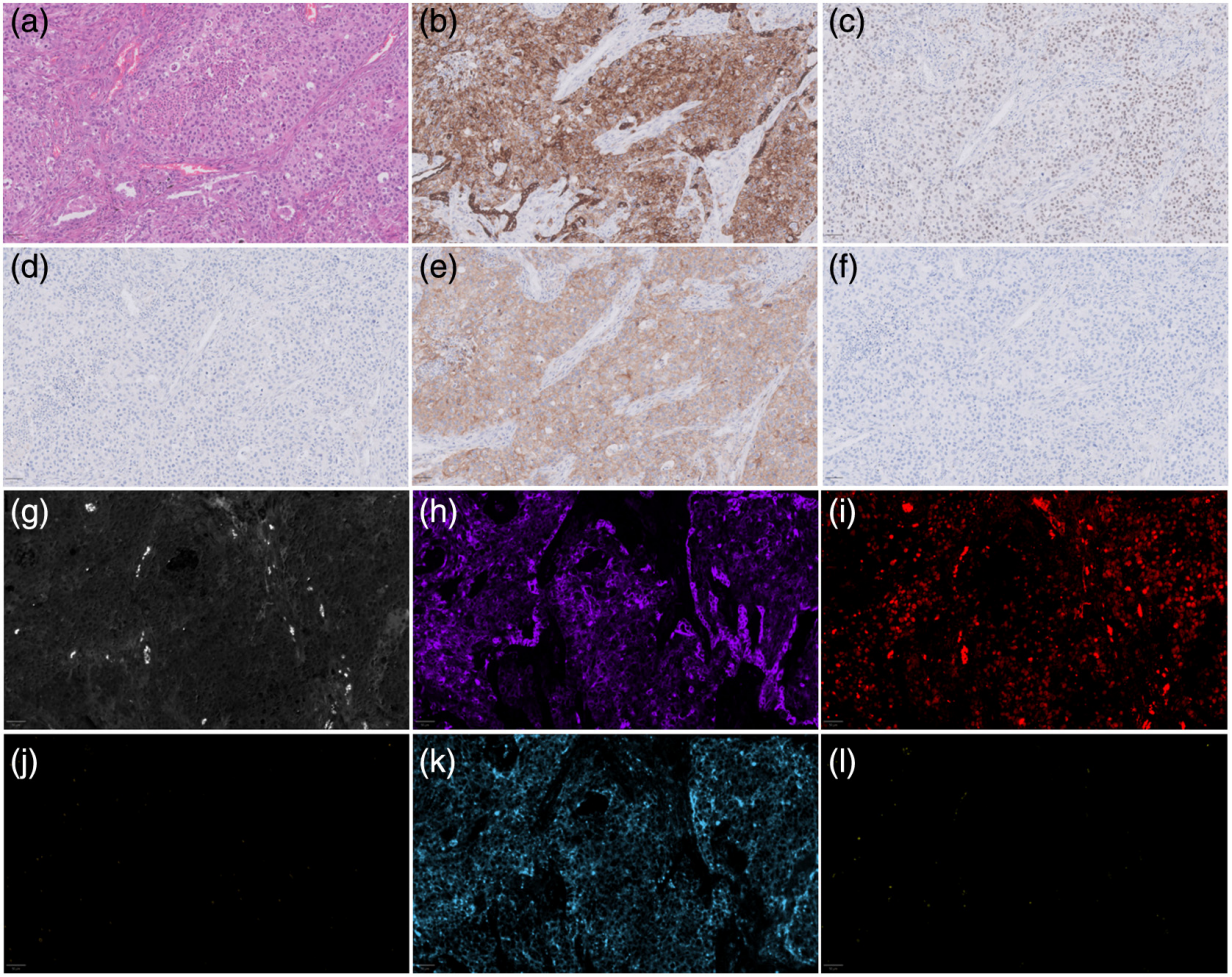
Comparison of single-plex IHC versus mIF unmixed channels data. Representative images of a selected tumor region from an NSCLC specimen, comparing conventional single-plex IHC and mIF acquired using the hyperspectral imaging system. Consecutive tissue sections of the same specimen were used. (a)–(f) Standard histopathology and IHC. (a) Hematoxylin and eosin. (b) Pan-cytokeratin (PanCK). (c) TTF1. (d) P40. (e) PD-L1. (f) ALK. (g)–(l) Corresponding mIF channels from the same region on a consecutive tissue section. (g) Tissue autofluorescence. (h) PanCK. (i) TTF1. (j) P40. (k) PD-L1. (l) ALK. The comparison illustrates similar and concordant staining patterns between single-plex IHC and multiplex imaging while enabling simultaneous visualization of all biomarkers within a single tissue section. Scale bar: 50  μm.

We next zoomed in on a specific tumor sub-area of the same specimen and compared the unmixed mIF data to the matching single-plex IHC data, produced using consecutive tissue sections. As illustrated in Fig. 5, both approaches yielded consistent staining profiles, with tumor cells demonstrating PanCK positivity [Figs. 5(b) and 5(h)], low-level TTF1 expression [Figs. 5(c) and 5(i)], PD-L1 positivity [Figs. 5(e) and 5(k)], and lack of both P40 [Figs. 5(d) and 5(j)] and ALK [Figs. 5(f) and 5(l)] expression.

In addition, a quantitative ROI-level comparison of staining intensities between chromogenic IHC and mIF using histologically matched consecutive tissue sections demonstrated strong concordance between the two methods for both PD-L1 ($R^{2}=0.86$) and PanCK ($R^{2}=0.89$; see Supplemental Fig. S3).

**Fig. 6:**
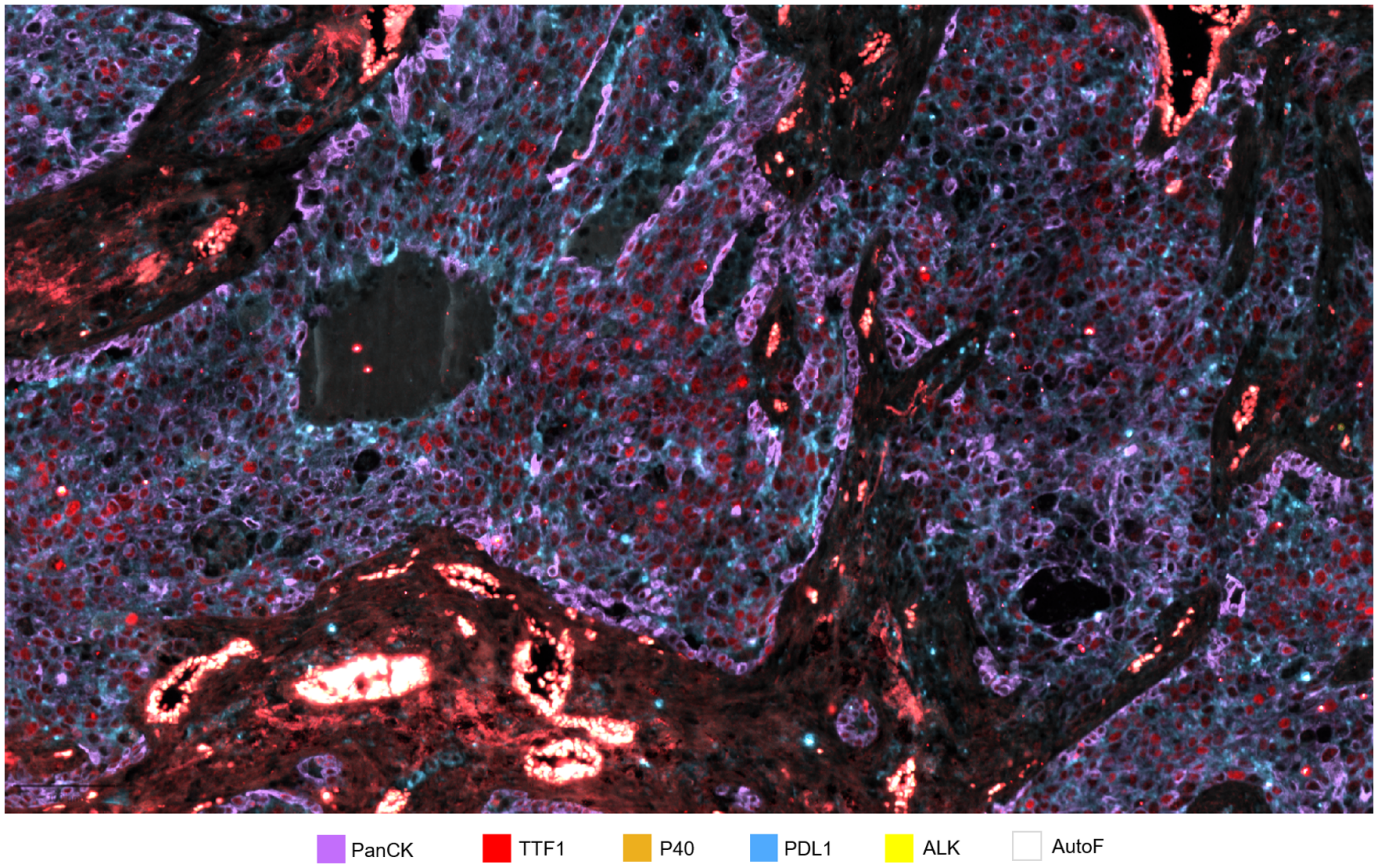
mIF visualization supporting diagnostic classification in an NSCLC specimen. Representative mIF image acquired using the hyperspectral imaging system, showing the combined spatial distribution of the following five biomarkers: PanCK, TTF1, P40, PDL1, and ALK within a single NSCLC tissue section. Pseudo-colors denote individual spectrally unmixed markers as indicated in the figure. Tissue autofluorescence (AutoF) is shown in a separate channel in white pseudo-color, demonstrating separation from the biomarker fluorescence channels. The composite biomarker profile enables integrated assessment of tumor identity and biomarker expression within tissue context, supporting diagnostic interpretation consistent with primary lung ADC with strong PD-L1 expression. The scale bar corresponds to 50  μm.

A key advantage of the multiplexed dataset is the ability to simultaneously visualize and interrogate all biomarkers within the same tissue section, enabling assessment of signal co-localization at the cellular and subcellular levels. This capability facilitates determination of tumor origin (here, epithelial), biomarker co-expression patterns, and estimation of the fraction of tumor cells expressing PD-L1, which may impact a patient’s eligibility for PD-1/PD-L1-directed immunotherapy. As shown in Fig. 6, the combined biomarker profile supports classification of this NSCLC case as a primary lung ADC with strong PD-L1 expression.

To further support reliable interpretation of multiplexed biomarker localization patterns, quantitative analyses were performed to assess the potential impact of spectral cross-talk under clinically relevant tissue conditions. Correlation analyses between spectrally neighboring fluorophore pairs associated with distinct subcellular compartments demonstrated absent to weak correlations, supporting robust spectral unmixing performance in clinical specimens (Supplemental Fig. S4).

**Fig. 7:**
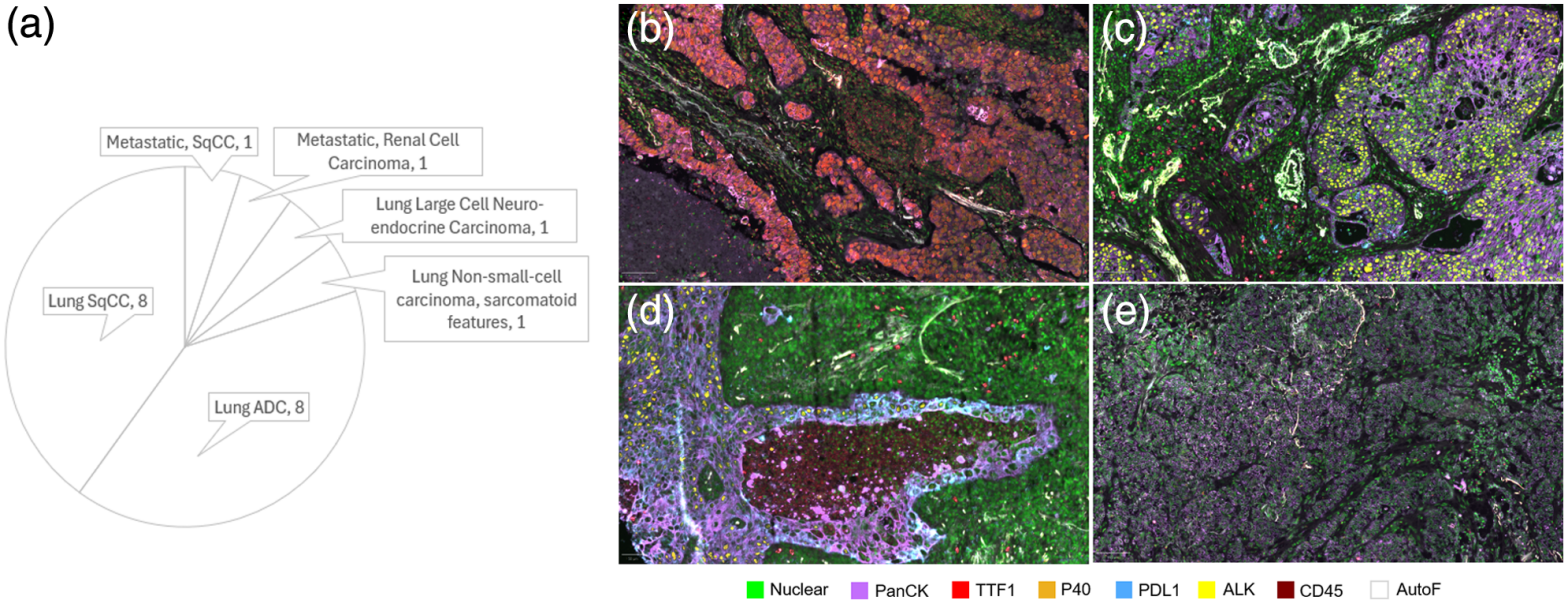
Composition of the retrospective TMA-based clinical validation cohort and representative mIF data from lung cancer cases. (a) Pie chart summarizing the diagnostic distribution of cases included in the retrospective TMA cohort analyzed in this pilot study. The cohort comprises predominantly primary NSCLC (eight Lung SqCC and eight lung ADC) and four non-NSCLC cases included to preliminarily evaluate assay performance. (b)–(e) Representative images of seven-plex mIF acquired using the hyperspectral imaging system, illustrating typical biomarker expression patterns observed across different diagnostic categories included in the TMAs. Pseudo-colors denote the following seven spectrally unmixed bio-markers used: PanCK, TTF1, P40, PDL1, ALK, CD45, and nuclear counter-staining, as also shown in the panel. (b) Primary lung ADC, characterized by PanCK positivity with TTF1 expression and undetectable P40 staining. (c) Primary lung SqCC, demonstrating PanCK positivity with P40 expression and undetectable TTF1 staining. (d) PD-L1-positive lung SqCC, showing co-localization of PD-L1- and PanCK-positive tumor cells. (E) Poorly differentiated carcinoma, as indicated by PanCK-positive TTF1/P40-double-negative tumor cells. Tissue autofluorescence (AutoF) is shown to illustrate background signal separation; 100-μm scale bars are shown in the lower left corner of each panel. The matching single-channel images of data shown here in panels (b)–(e) are presented in Supplemental Figs. S6–S9, respectively.

To evaluate diagnostic concordance at the cohort level, pathologist-curated TMA slides containing a range of thoracic pathologies were analyzed. A sub-cohort of 20 cases, composed of 16 pre-diagnosed NSCLC samples and 4 non-NSCLC thoracic pathologies, was selected for analysis [Fig. 7(a)]. Multiplexed data images of representative clinical cases and categories from selected TMA cores are shown in Figs. 7(b)–7(e). Notably, all diagnoses derived from the single-slide seven-plex mIF datasets were concordant with the corresponding reference diagnoses established using standard single-plex chromogenic IHC assays across the evaluated cohort.

## 4 Discussion and Conclusion

In this study, we introduced a hyperspectral imaging platform enabling mIF-based detection of cancer biomarkers, designed with an emphasis on potential clinical applicability in routine diagnostic workflows. By integrating a monolithic Sagnac interferometer with Fourier-transform-based spectral reconstruction, the system provides high spectral resolution and mechanical stability, while its simplified optical architecture supports future translation into clinical pathology laboratories.

Clinical NSCLC excision specimens were initially used during assay development and representative-case evaluation, followed by a retrospective clinical validation pilot study using a pathologist-curated TMA cohort. Together, these analyses demonstrated accurate, high-content biomarker profiling from a single tissue section. This capability is relevant to diagnostic workflows of various cancers and particularly to lung cancer diagnostics, where biopsy material is frequently limited. Simultaneous assessment of multiple biomarkers on the same slide conserves tissue while preserving spatial context, enabling co-localization analysis that is increasingly important for immune phenotyping and characterization of the tumor microenvironment. In addition, integrated visualization of multiple biomarker distributions within a single tissue section may facilitate more comprehensive interpretation of complex pathological features. Importantly, diagnoses derived from the multiplexed datasets were fully concordant with reference clinical IHC across the evaluated cases. Although the present cohort size limits direct assessment of clinical outcome prediction, the multiplexed panel enables resolution of diagnostically relevant biomarker combinations—such as PD-L1 expression in tumor versus immune cells—that are known to influence therapeutic decision-making, including eligibility for PD-1/PD-L1 immune checkpoint inhibitor-based therapy. These findings support the potential role of multiplexed imaging as a component of precision oncology workflows.

Beyond lung cancer, the platform may be adaptable to additional tumor types, tissue origins, and diverse multiplexed biomarker panels, potentially extending its utility across broader pathology applications. Such adaptability is further supported by the demonstrated use of clinically validated antibody clones, established signal amplification methods, and a mechanically stable monolithic interferometer architecture, which together may facilitate integration with existing histopathology workflows and laboratory practices.

Several limitations warrant consideration. Although the system demonstrated promising performance on retrospective specimens, prospective clinical validation in real-world diagnostic workflows will be required to establish its clinical utility in routine diagnostic practice. Although the current implementation supports seven markers, future work will focus on expanding panel size while preserving spectral separation and image quality. This may be achieved through the incorporation of additional excitation wavelengths and optimization of existing system capabilities, with the goal of enabling visualization of larger biomarker panels. The current implementation is associated with relatively long acquisition times (∼10  h per 15  mm×15  mm tissue section at 20× magnification), which may limit integration into routine high-throughput clinical workflows. Notably, the present system configuration has not yet been optimized for scanning throughput, inter-device communication, or computational efficiency. Hence, future optimization of hardware and graphics processing unit (GPU)-accelerated computational workflows is expected to substantially reduce acquisition and processing times. Clinical-scale deployment will also require efficient data management, storage, and computational infrastructure for high-volume hyperspectral imaging datasets. In addition, integration with automated image analysis pipelines for cell segmentation and quantitative biomarker assessment will be important for streamlining data interpretation and reducing inter-observer variability in clinical settings.

Overall, these results highlight the potential of hyperspectral multiplexed imaging to address key limitations of traditional single-plex IHC. With continued technical refinement and clinical validation, this approach may improve tissue utilization and facilitate integrated spatial biomarker assessment across oncology and broader pathology applications.

## Supplementary Material

10.1117/1.JBO.31.7.076504.s01

10.1117/1.JBO.31.7.076504.s02

10.1117/1.JBO.31.7.076504.s03

10.1117/1.JBO.31.7.076504.s04

10.1117/1.JBO.31.7.076504.s05

10.1117/1.JBO.31.7.076504.s06

10.1117/1.JBO.31.7.076504.s07

10.1117/1.JBO.31.7.076504.s08

10.1117/1.JBO.31.7.076504.s09

## Data Availability

The data that support the findings of this study are available from the corresponding author upon reasonable request.
