# Supplementary figures and images for: Hyperspectral imaging system for multiplexed cancer marker detection: design and clinical evaluation
Source: J Biomed Opt. 2026 Jul 9;31(7):076504. doi: 10.1117/1.JBO.31.7.076504 (PMC13349328; doi:10.1117/1.JBO.31.7.076504)

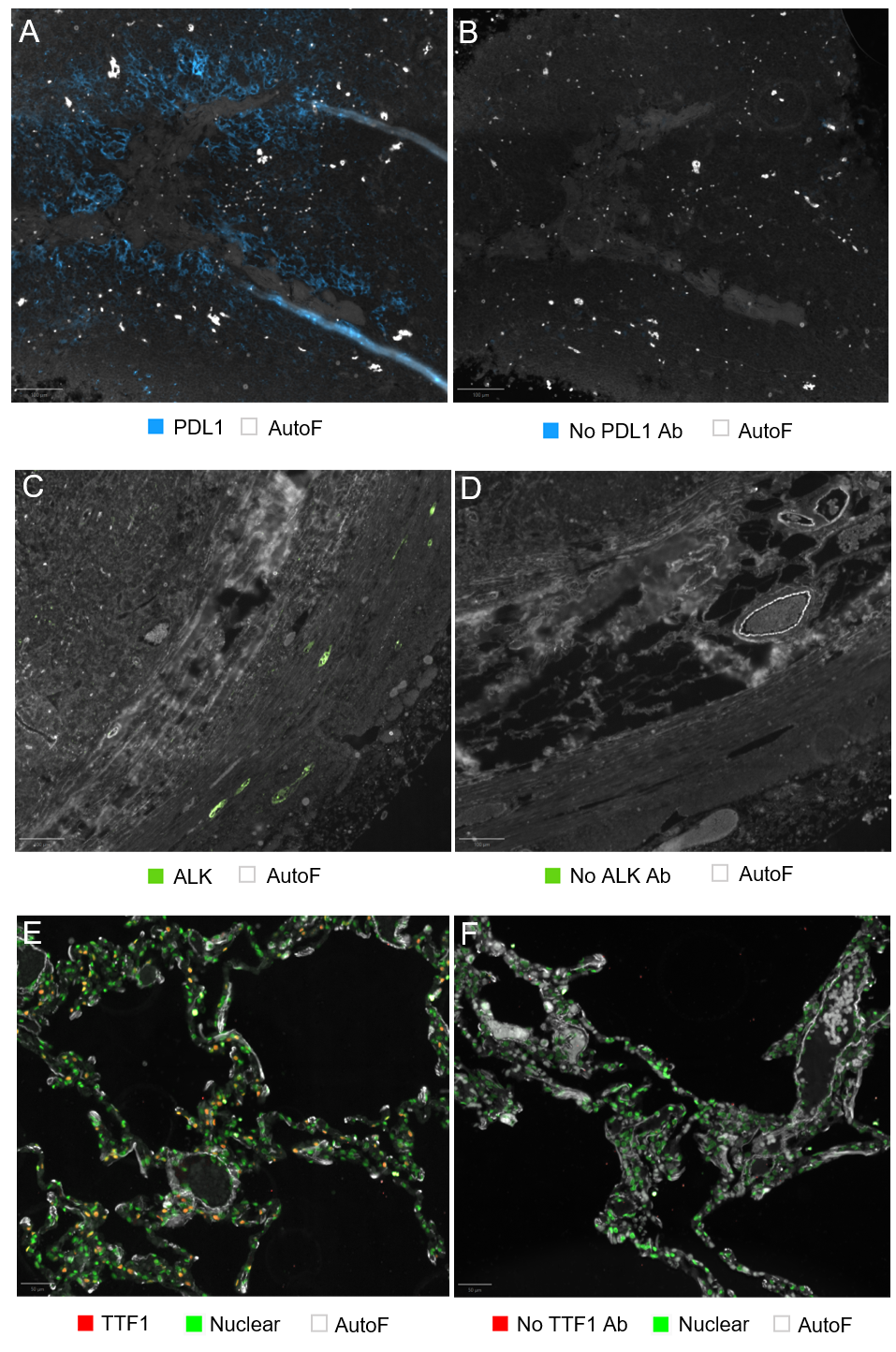

Supplement: Supplementary file 1 [file JBO_031_076504_SD001.png]

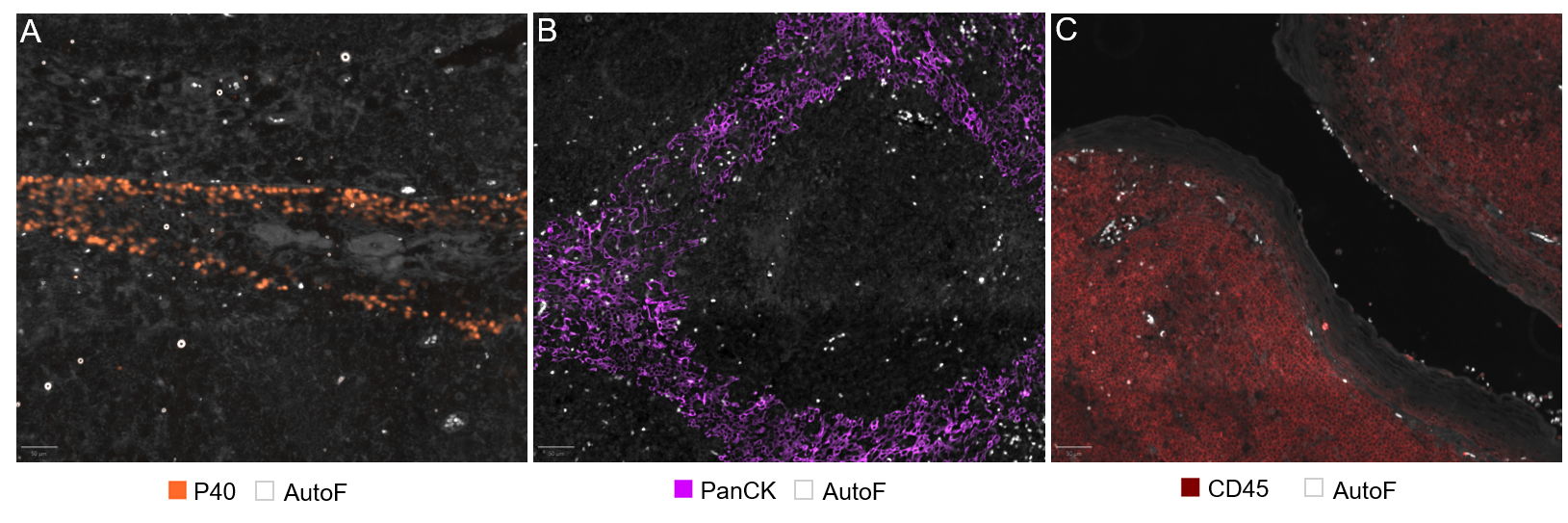

Supplement: Supplementary file 2 [file JBO_031_076504_SD002.png]

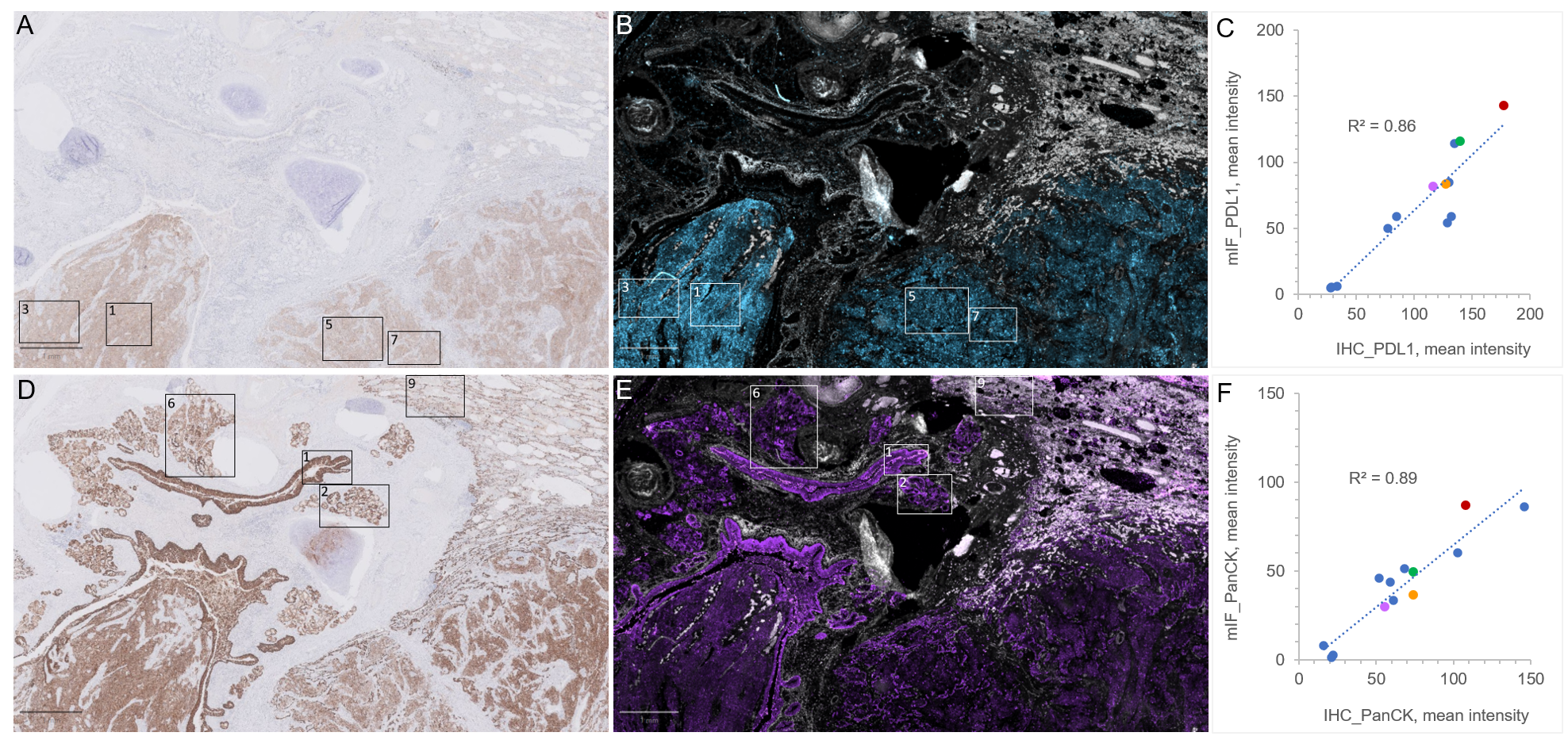

Supplement: Supplementary file 3 [file JBO_031_076504_SD003.png]

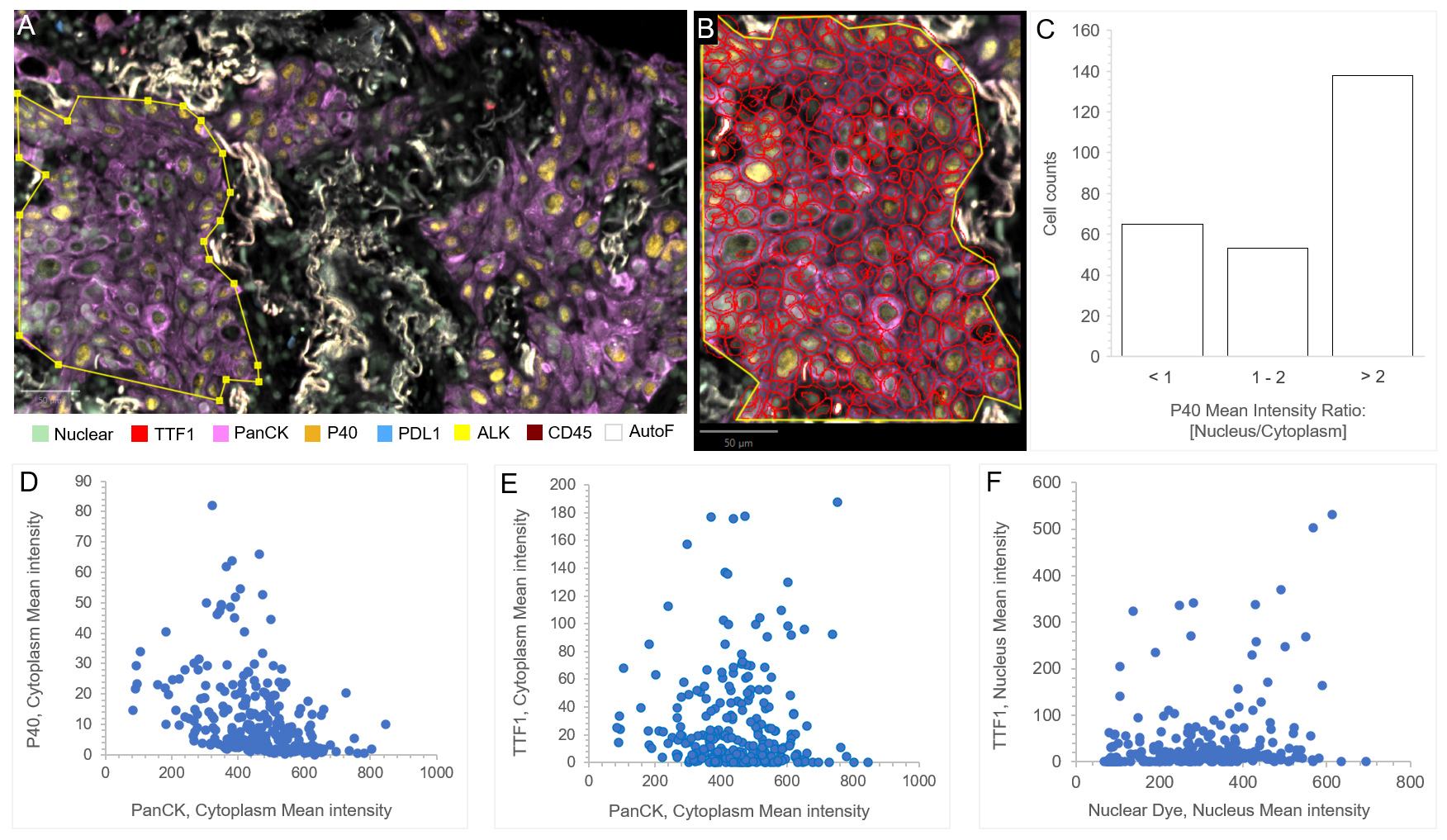

Supplement: Supplementary file 4 [file JBO_031_076504_SD004.png]

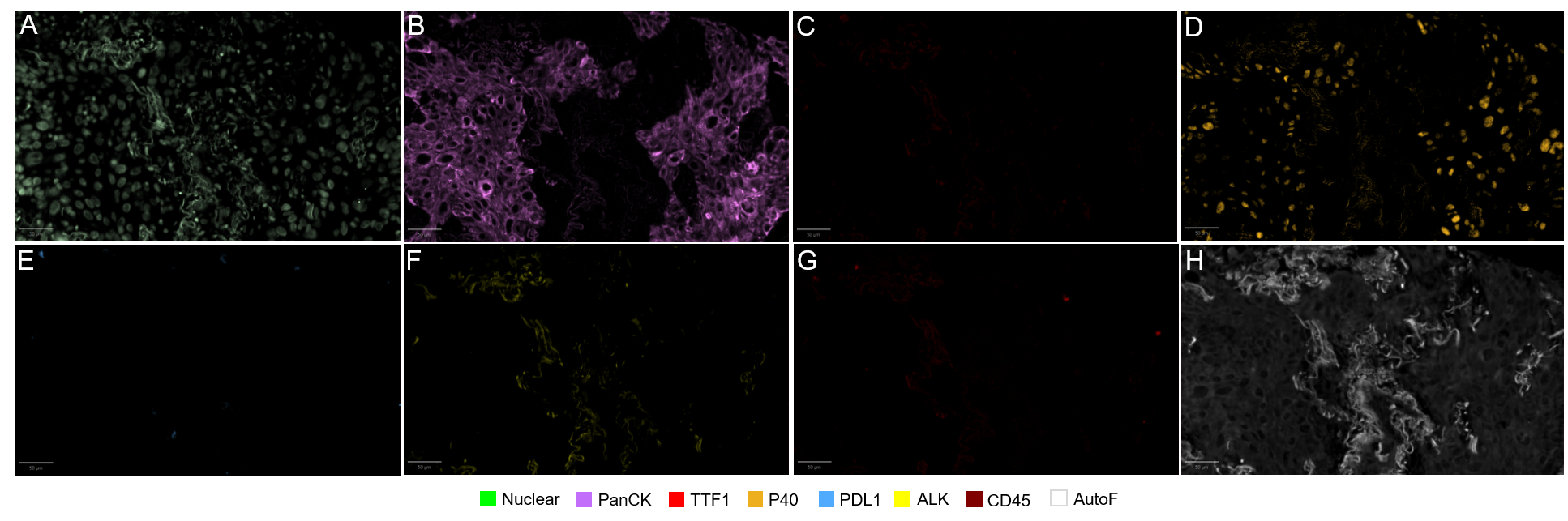

Supplement: Supplementary file 5 [file JBO_031_076504_SD005.png]

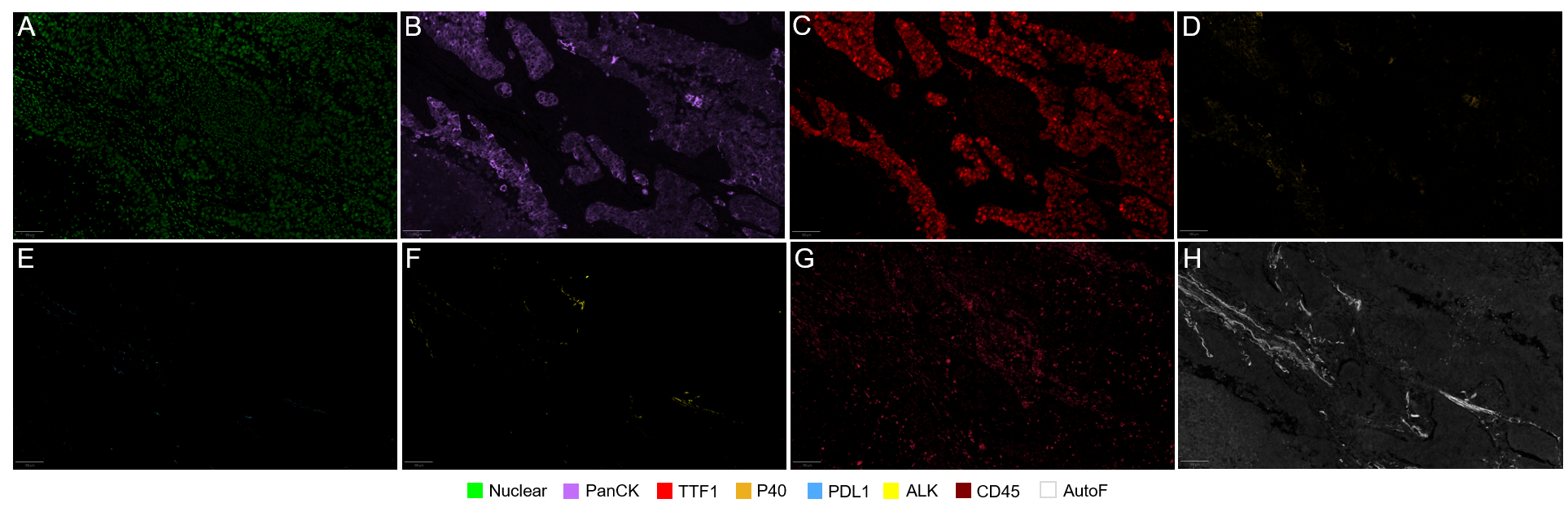

Supplement: Supplementary file 6 [file JBO_031_076504_SD006.png]

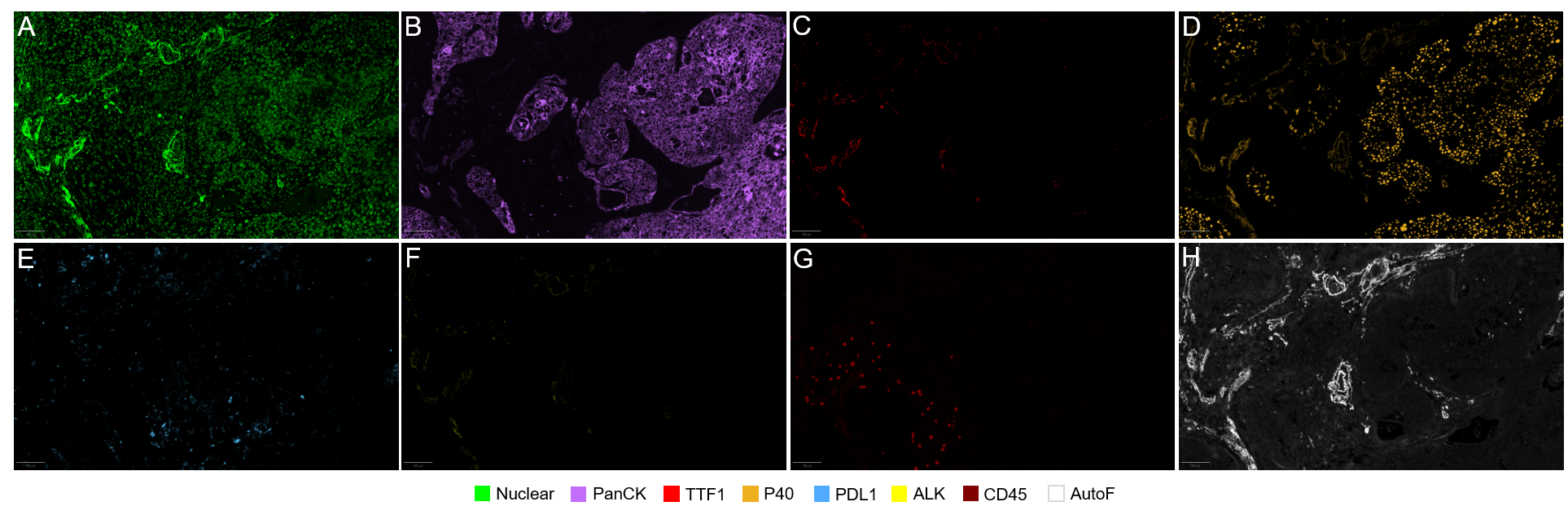

Supplement: Supplementary file 7 [file JBO_031_076504_SD007.png]

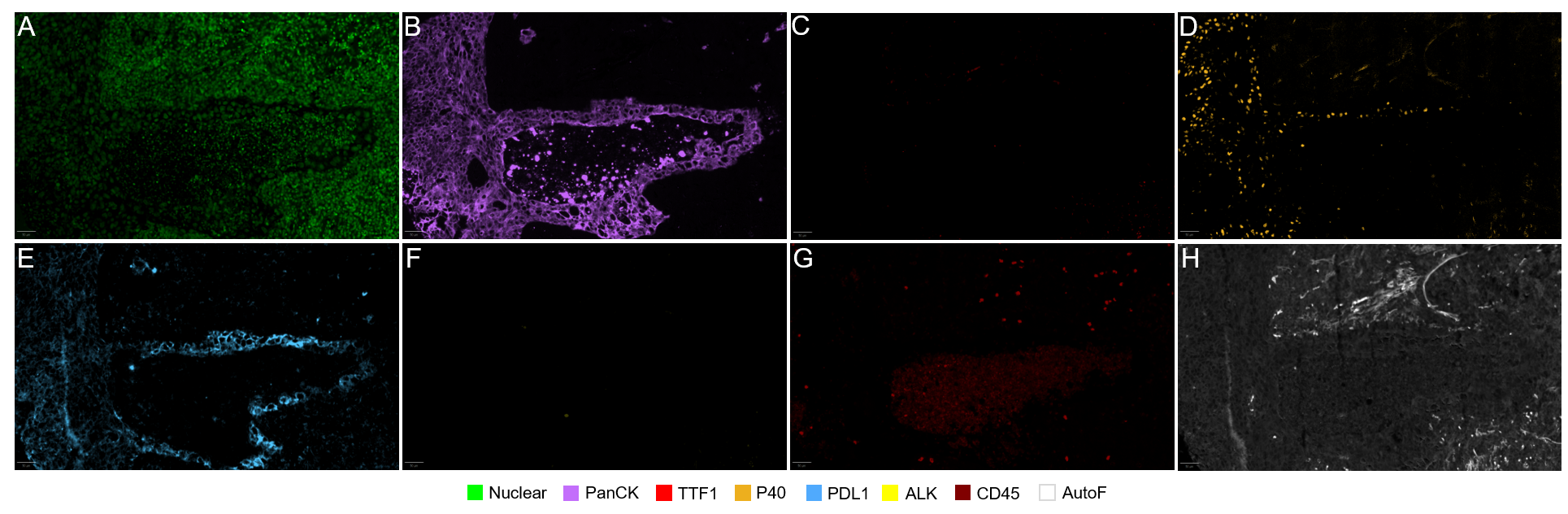

Supplement: Supplementary file 8 [file JBO_031_076504_SD008.png]

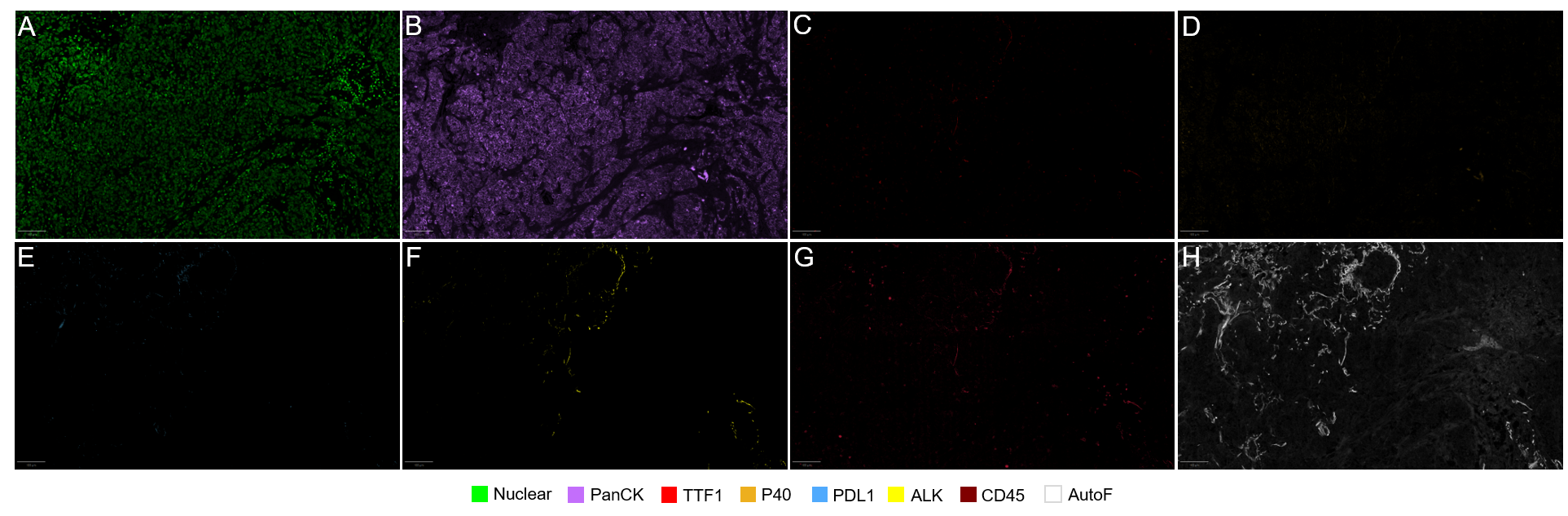

Supplement: Supplementary file 9 [file JBO_031_076504_SD009.png]
